# Physiological and transcriptomic response to methyl-coenzyme M reductase limitation in *Methanosarcina acetivorans*

**DOI:** 10.1128/aem.02220-23

**Published:** 2024-06-25

**Authors:** Grayson L. Chadwick, Gavin A. Dury, Dipti D. Nayak

**Affiliations:** 1 Department of Molecular and Cell Biology, University of California, Berkeley, California, USA; University of Nebraska-Lincoln, Lincoln, Nebraska, USA

**Keywords:** methanogen, methane, *Methanosarcina*

## Abstract

Methyl-coenzyme M reductase (MCR) catalyzes the final step of methanogenesis, the microbial metabolism responsible for nearly all biological methane emissions to the atmosphere. Decades of biochemical and structural research studies have generated detailed insights into MCR function *in vitro*, yet very little is known about the interplay between MCR and methanogen physiology. For instance, while it is routinely stated that MCR catalyzes the rate-limiting step of methanogenesis, this has not been categorically tested. In this study, to gain a more direct understanding of MCR’s control on the growth of *Methanosarcina acetivorans,* we generate a strain with an inducible *mcr* operon on the chromosome, allowing for careful control of MCR expression. We show that MCR is not growth rate-limiting in substrate-replete batch cultures. However, through careful titration of MCR expression, growth-limiting state(s) can be obtained. Transcriptomic analysis of *M. acetivorans* experiencing MCR limitation reveals a global response with hundreds of differentially expressed genes across diverse functional categories. Notably, MCR limitation leads to strong induction of methylsulfide methyltransferases, likely due to insufficient recycling of metabolic intermediates. In addition, the *mcr* operon is not transcriptionally regulated, i.e., it is constitutively expressed, suggesting that the overabundance of MCR might be beneficial when cells experience nutrient limitation or stressful conditions. Altogether, we show that there is a wide range of cellular MCR concentrations that can sustain optimal growth, suggesting that other factors such as anabolic reactions might be rate-limiting for methanogenic growth.

**IMPORTANCE:**

Methane is a potent greenhouse gas that has contributed to ca. 25% of global warming in the post-industrial era. Atmospheric methane is primarily of biogenic origin, mostly produced by microorganisms called methanogens. Methyl-coenzyme M reductase (MCR) catalyzes methane formatio in methanogens. Even though MCR comprises ca. 10% of the cellular proteome, it is hypothesized to be growth-limiting during methanogenesis. In this study, we show that *Methanosarcina acetivorans* cells grown in substrate–replicate batch cultures produce more MCR than its cellular demand for optimal growth. The tools outlined in this study can be used to refine metabolic models of methanogenesis and assay lesions in MCR in a higher-throughput manner than isolation and biochemical characterization of pure protein.

## INTRODUCTION

Methyl coenzyme M reductase (MCR) catalyzes the final step of methane production in methanogenic archaea (1). The active enzyme consists of three subunits in an α_2_β_2_γ_2_ stoichiometry that is present in very high abundance in the cytosol. The operon encoding MCR dominates methanogen transcriptomes, where it is invariably found to be one of the most abundant mRNAs (2), and MCR accounts for roughly 10% of the cytoplasmic proteome (1, 3). MCR is also the first step of anerobic methane oxidation in anerobic methanotrophic archaea, where it is found in similarly high levels in transcriptomes (4, 5) and proteomes (6, 7). Despite its abundance, the isolation and biochemical characterization of active MCR is challenging. The active site contains a nickel porphyrin cofactor (F430) that is significantly oxygen-sensitive, and even when the enzyme is purified in the absence of oxygen, it can enter inactive product-inhibited states. While a few protocols have been developed to purify the active enzyme, or re-activate inactive states, from *Methanothermobacter marburgensis*, these are not broadly applicable to other methanogens, especially strains with sophisticated tools for genetic manipulation, such as *Methanococcus maripaludis* or *Methanosarcina* spp. (1). Based on these challenges, even straightforward experiments to measure kinetic parameters such as substrate affinities (K_m_) or turnover rate (k_cat_) have taken decades of effort from multiple research groups and have not been expanded to cover any significant phylogenetic diversity.

In contrast to the advances made in understanding the biochemical properties of the *M. marburgensis* MCR in isolation, holistic questions that address the biogenesis and function of MCR, likely mediated by an interaction with other proteins, as well as with methanogen physiology more broadly, have received significantly less attention. We know very little about the proteins involved in the assembly, activation, and degradation of MCR *in vivo*. Similarly, regulatory processes that control the expression of MCR in response to environmental cues such as resource availability or stress response have barely been studied. The only system where regulation has been investigated is the differential expression of the two isoforms of MCR present in *Methanothermobacter* spp*.* (8) ; however, most methanogens carry only a single copy of MCR (1). How, and to what extent, methanogens control the amount of MCR and its activity when conditions are unfavorable is an understudied but important question due to the growing interest in inhibiting methanogenesis for reducing methane emissions, as well as using methanogens as a chassis for bioengineering, wherein major metabolic end-products in addition to methane are desired (9).

Recent advances in genetic tools available for the study of methanogens have facilitated targeted mutagenesis of genes encoding hypothesized accessory proteins of MCR and provided an avenue to investigate their cellular functions. Using this approach, the identity and function of many MCR-associated proteins involved in the installation of post-translational modifications and insertion of F430 have been successfully studied (10–12). One surprising outcome of these genetic studies is that the loss of highly conserved MCR-associated proteins often has minimal or no significant effects on cell growth, even though, to the best of our knowledge, MCR is essential in all methanogenic archaea (10–12). A possible explanation for these results is that MCR is not rate-limiting for growth in substrate-replete batch cultures. Therefore, even mutations that result in a substantial detrimental impact to MCR function may be tolerated without a notable growth defect, as has been recently suggested in (1).

No direct evidence linking MCR abundance to growth and methanogenesis is currently available, and there are differing views in literature. A recent study used a kinetic and stoichiometric hybrid approach to model the growth of *Methanosarcina barkeri* on methanol (13). They showed that MCR has a high control coefficient (of ~0.9) during growth on high methanol concentrations (> 15 mM), i.e., small changes in MCR levels will have a dramatic impact on growth rate under typical batch-culture conditions. This model as well an older kinetic model for *Methanosarcina acetivorans* (14) corroborate a long-standing hypothesis that MCR is a rate-limiting enzyme during methanogenic growth (8, 15). Alternately, other studies have targeted F430 biosynthesis, either through the omission of nickel in the growth medium or through the addition of levulinic acid, which inhibits porphyrin biosynthesis (3, 16, 17). These studies show that a modest decrease in F430 abundance has no effect on cell growth, and only a drastic reduction in F430 levels, by fivefold or more, leads to growth defects and can alter subcellular localization of MCR. Taken together, these studies are consistent with the notion that MCR is present in excess in nickel-replete medium typically used for laboratory cultivation of methanogens (3). One caveat of these studies is that nickel and porphyrins are present in many other bioenergetic enzymes essential for methanogenesis; hence, it is difficult to attribute any observed growth phenotypes entirely to MCR. Additionally, carbon monoxide supplementation inhibits methanogenesis in *Methanosarcina acetivorans,* and growth under these conditions produces large quantities of acetate, formate, and methylsulfides and only a small amount of methane (18–20). Acetogenic growth of *M. acetivorans* can be further amplified in mutants where methane production is nearly completely abolished (21). While it has been suggested that carbon monoxide is partially inhibitory to MCR (18), it is not clear that there is a biochemical basis for this notion. Notably, even under conditions where methane production is an insignificant portion of catabolism, MCR remains essential and cannot be deleted (21).

Based on the current literature, it is unclear if MCR is present in excess during laboratory growth, and if so, why such a substantial portion of their proteome and transcriptome might be allocated to it, especially when there is a well-established precedent that methanogens have elaborate mechanisms for modifying the expression of other metabolic genes (22). Hence, to better understand this interplay between MCR abundance and cell growth, we investigated the physiology of the genetically tractable strain *M. acetivorans* carrying an inducible MCR operon. Our results clearly demonstrate that wild-type expression of MCR far exceeds the cellular demand and also that MCR-limited growth is indeed possible at significantly lower levels of expression. Under these MCR-limiting conditions, there is a global transcriptional shift that alters the expression of hundreds of genes involved in a variety of cellular processes beyond methanogenesis.

## RESULTS AND DISCUSSION

### Halting the transcription of the MCR operon leads to linear growth

**Fig 1:**
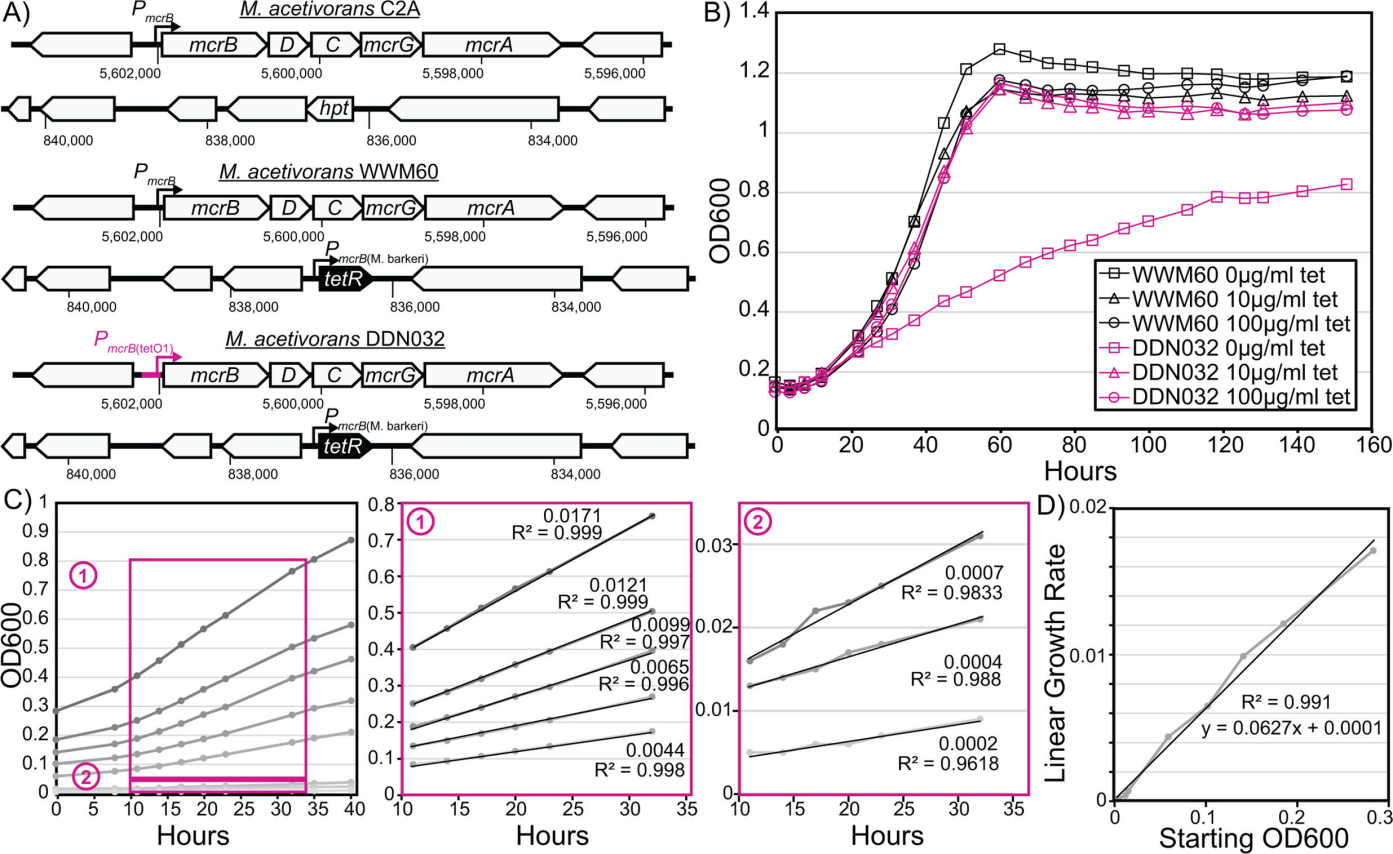
Mutant construction and growth characteristics. (**A**) Genotype of wild-type *M. acetivorans* C2A, the strain capable of inducible expression via the chromosomal integration of *tetR* (WWM60) and the inducible MCR strain generated in this study (DDN032). (**B**) Growth characterization of DDN032 on high-salt trimethylamine media demonstrates a linear growth phenotype upon transfer into media lacking tetracycline (representative growth curves shown). (**C**) Linear growth curves of DDN032 in tetracycline-free media at various starting optical densities. Boxes 1 and 2 show details of regions used for linear regression calculations. Slopes and R^2^ values are shown beside each line in boxes 1 and 2. (**D**) The slopes of the linear regressions from panel C are plotted as a function of the starting optical density, demonstrating a strong linear relationship.

*M. acetivorans* strain WWM60 encodes a *tetR* gene under the control of the *mcrB* promoter from *Methanosarcina barkeri* Fusaro in place of the *hpt* locus (MA0717 or MA_RS03755), enabling tetracycline-based control of gene expression and protein production (23). We used WWM60 as the genetic background to introduce a *tetO1* operator site in the promoter of the *mcr* operon and generated the mutant strain DDN032, wherein the expression of the *mcr* operon can be titrated by the addition of tetracycline to the growth medium (Fig. 1A). Whole-genome resequencing of DDN032 verified the desired chromosomal change as well as the absence of any suppressor mutation(s) or off-target effects due to CRISPR editing (Fig. S1). Tetracycline-dependent expression of MCR in DDN032 was also confirmed by measuring transcript and protein levels at different tetracycline concentrations (see section titled “Effect of tetracycline on transcriptome and MCR protein abundance” below). Unless specified, this strain was passaged in media containing 100 µg/m tetracycline, which is considered full induction of the P*mcrB*(*tetO1*) promoter (23).

To determine the effect of MCR expression on growth, we inoculated exponential phase cultures of WWM60 and DDN032 (previously grown in medium with 100 µg/mL tetracycline and washed in tetracycline-free media three times) in media containing 0, 10, or 100 µg/mL tetracycline with trimethylamine as their carbon source. DDN032 grew indistinguishably from WWM60 in medium supplemented with 10 or 100 µg/mL tetracycline; however, there was a clear distinction between the two strains in the absence of tetracycline (Fig. 1B). Under these conditions, DDN032 has a biphasic growth curve starting with a short phase of exponential growth (corresponding to approximately one doubling), followed by linear growth. Linear growth can result from the dilution of a growth-limiting substance upon cell division such that the two daughter cells will have half the growth rate of the parental cell (24). We hypothesize that the linear growth observed in this study is due to continuous dilution of growth-limiting amounts of MCR protein once MCR production has been halted. A short period of exponential growth before onset of linear growth has previously been observed for nickel limitation in *Methanothermobacter thermautotrophicus* (3).

If MCR limitation leads to linear growth of DDN032, then based on prior art (24), the rate of linear growth would be directly proportional to the amount of MCR (the limiting substance) in the inoculum. To test this hypothesis, cultures of DDN032 grown with 100 µg/mL tetracycline were washed thrice and inoculated into tubes without tetracycline at varying starting optical densities (Fig. 1C). Within 10 hours, cultures entered linear growth in which the rate of increase in optical density was entirely dependent on the starting optical density, consistent with the prediction of MCR being the limiting resource, giving rise to linear growth (Fig. 1D). We were able to observe slow linear growth for weeks in cultures seeded with a low inoculum; however, after prolonged incubation, escape mutations (i.e., mutations that restore growth in the absence of tetracycline) were commonly observed due to the inactivation of *tetR* by endogenous transposon insertion (Fig. S2).

### Measuring the expression threshold for aberrant growth due to MCR limitation

**Fig 2:**
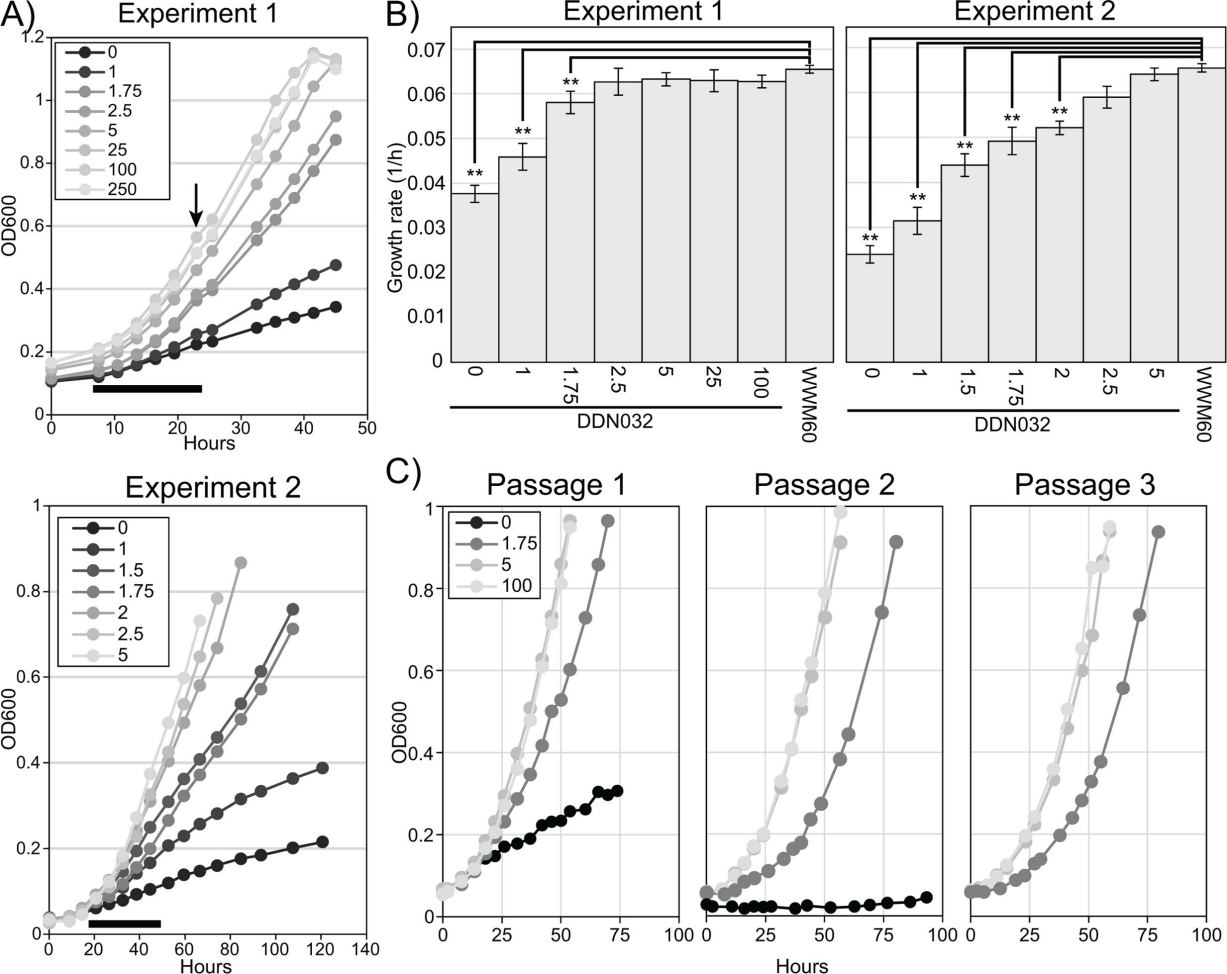
Effect of tetracycline on the growth rate. (**A**) Cultures of DDN032 pre-grown in media with 100 µg/mL tetracycline were washed thrice and inoculated into Balch tubes with eight different concentrations of tetracycline (μg/mL) in quintuplicate (Experiment 1, top) and seven different concentrations in quadruplicate (Experiment 2, bottom). Representative growth curves are shown here; all growth data can be found in Fig. S3 and S4. The black bar below growth curves shows the time range where growth data were calculated for panel B, and the arrow indicates in Experiment 1 where three of five replicates were sacrificed for RNA extraction (see Fig. 3 and 4). (**B**) Growth rates for DDN032 and WWM60 in the conditions indicated. Error bars indicate standard deviations of quintuplicate cultures for Experiment 1 and quadruplicate cultures for Experiment 2. Growth rates were calculated from exponential fits in the region shown by a black bar in panel A, as discussed in the text. Note: growth for WWM60 with 100 µg/mL tetracycline was only carried out in Experiment 1. Growth rates that are significantly different than those of WWM60 with 100 µg/mL tetracycline ANOVA and Tukey’s Honest Significant Difference test are indicated (** *P* value ≤ 0.01). (**C**) Sequential passaging of DDN032 at 1.75 µg/mL tetracycline reveals reproducibly slower exponential growth (also see Fig. S5).

With clear evidence that complete MCR suppression leads to linear growth in *M. acetivorans*, we sought to determine the minimum amount of tetracycline necessary for wild-type levels of growth. To determine this induction threshold, DDN032 was inoculated into media supplemented with a series of tetracycline concentrations. In two separate experiments, growth was analyzed in quintuplicate cultures with a wide range of tetracycline concentrations (Experiment 1) or quadruplicate cultures with a fine range of tetracycline concentrations (Experiment 2), all with methanol as the carbon source (Fig. 2A; Fig. S3 and S4). For quantitative comparisons between all growth conditions, we calculated growth rates based on exponential fits of the initial one to two doublings (Fig. 2B). In both experiments, when growth rates of DDN032 were compared to those of WWM60, there was no significant differences in growth at tetracycline concentrations ≥ 2.5 µg/mL, whereas a detectable growth defect was observed at tetracycline concentrations ≤ 1.75 µg/mL in Experiment 1 and ≤2 µg/mL in Experiment 2.

**Fig 3:**
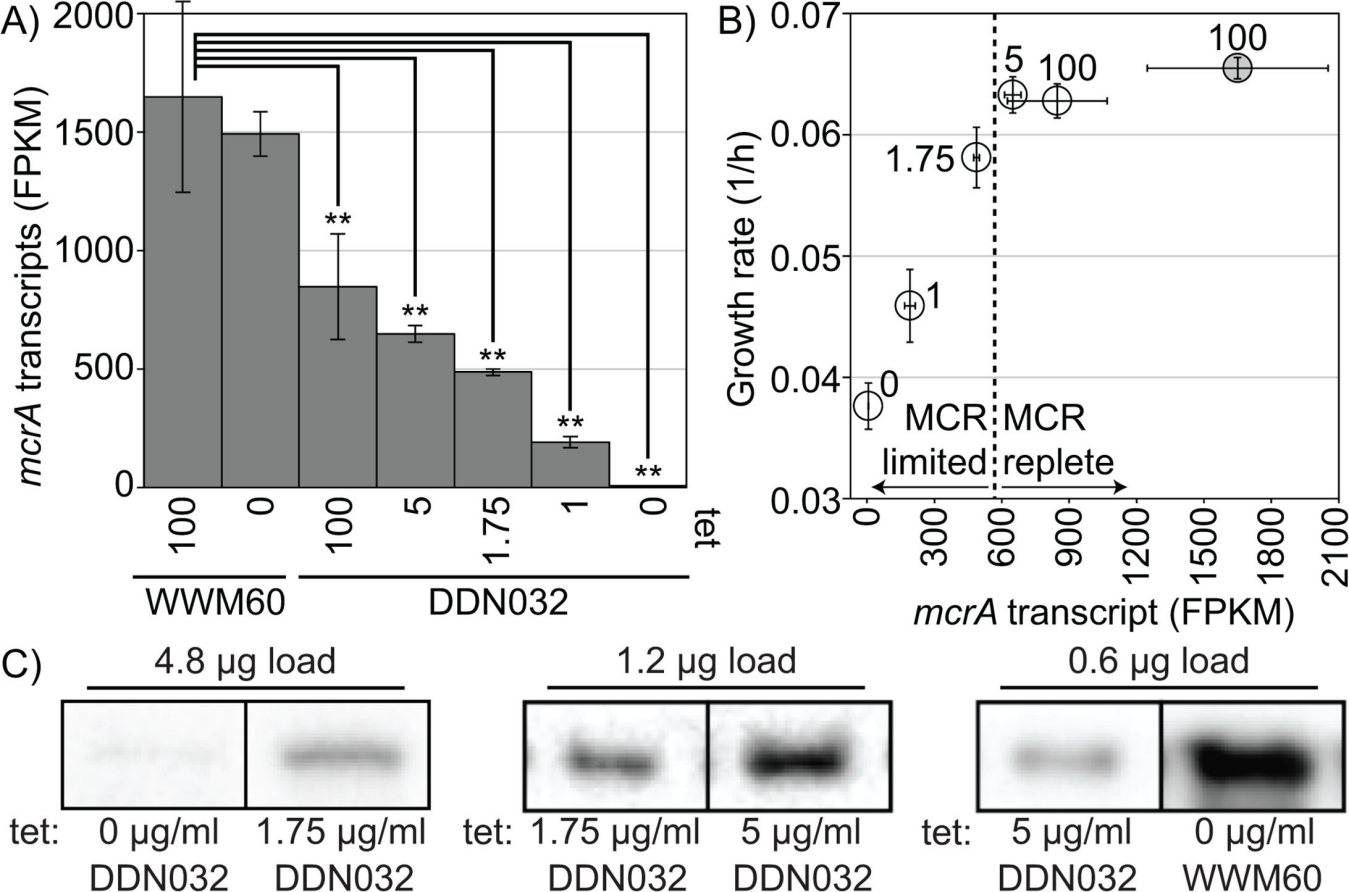
MCR transcript and protein response to tetracycline concentration. Cultures grown at 0, 1, 1.75, 5, or 100 µg/mL tetracycline (DDN032) or 0 and 100 µg/mL (WWM60) were used for RNA sequencing. (**A**) Transcriptional response of the *mcrA* gene to tetracycline concentration in WWM60 and DDN032. Transcript abundances that are significantly different than those of WWM60 with 100 µg/mL tetracycline by ANOVA and Tukey’s Honest Significant Difference test are indicated (** *P* value ≤ 0.01). Error bars represent standard deviations of biological triplicates. (**B**) Growth rates of cultures vs the *mcrA* transcript level, open circles DDN032, and shaded circle WWM60 with 100 µg/mL tetracycline. Error bars represent standard deviations of biological triplicates, the dashed line represents an apparent switch between MCR replete conditions and MCR limited growth, and numbers next to the points indicate tetracycline concentration (μg/mL). (**C**) Representative bands from immunoanalysis with antibodies raised against McrA from *Methanosarcina acetivorans*. Pairwise comparisons between MCR abundance at different levels of tetracycline concentrations (listed below each band) in DDN032 and WWM60. Bands with the same total protein load are noncontiguous selections from the same blot. Comparisons between protein concentrations should only be made for bands derived from the same blot. Blots showing relative concentrations by dilution to extinction are available in the supplement (Fig. S7). Note: for DDN032 grown with 1 µg/mL, only two replicates were available for RNA sequencing.

**Fig 4:**
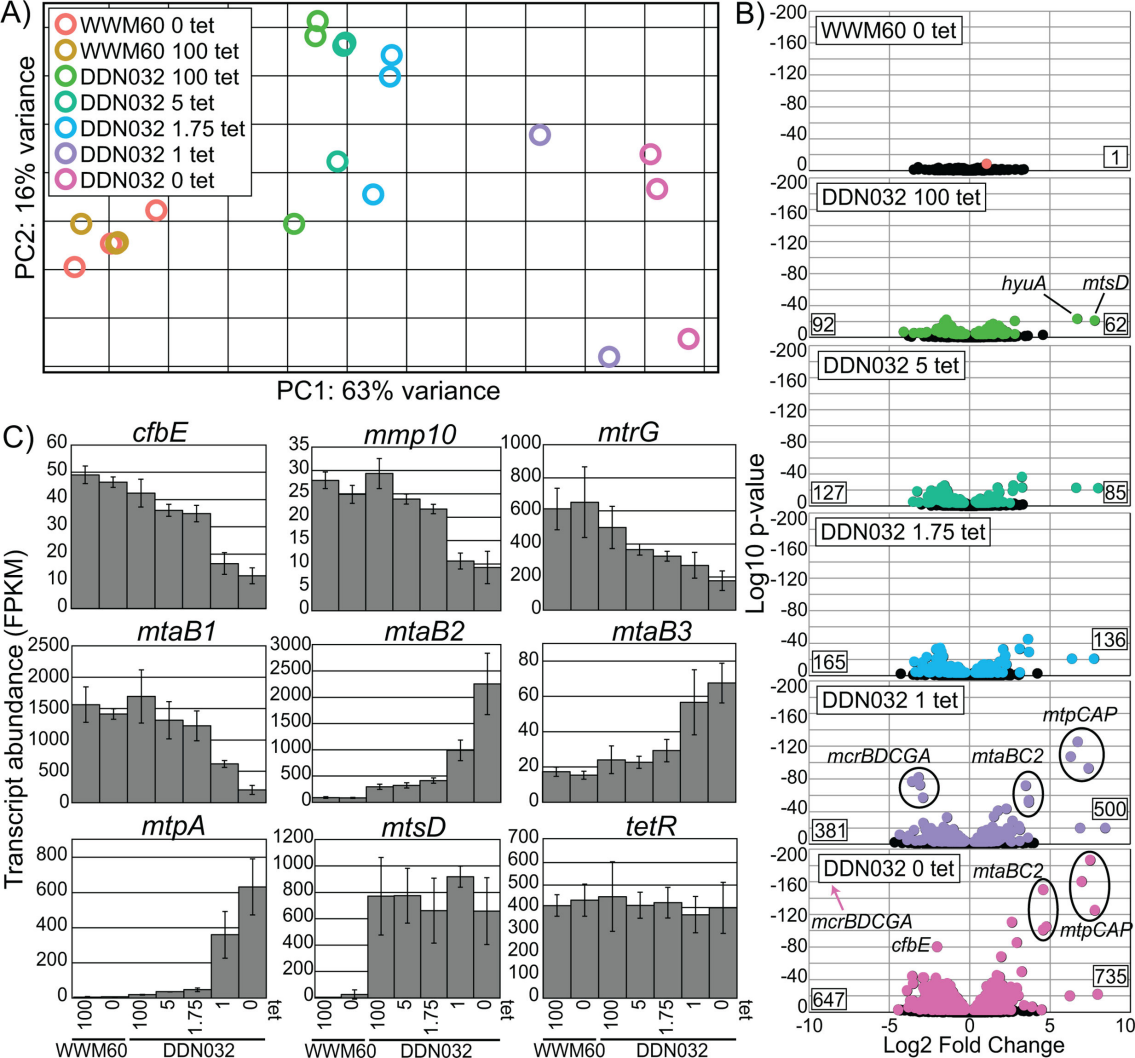
Global transcriptomic response to MCR limitation. (**A**) Global transcriptomic response to different tetracycline concentrations captured by principal component analysis. (**B**) Volcano plots depicting the log2-fold change for genes of all conditions compared to WWM60 grown on 100 µg/mL tetracycline. Negative values indicate lower expression relative to WWM60 on 100 µg/mL tetracycline. All genes significantly differentially expressed based on multiple comparison adjusted *P*-value cutoff of 0.01 are shown in color and nonsignificant genes in black. Numbers on the bottom left and right corners of each plot represent the total number of genes significantly down or upregulated, respectively. Certain genes exhibiting strong fold-change differences mentioned in the text are highlighted. (**C**) Specific gene expression profiles as a function of tetracycline concentration. Error bars represent standard deviations of biological triplicates. For *mtpA* and *mtsD,* all DDN032 conditions are significantly upregulated compared to WWM60 at a multiple comparison corrected *P*-value of <0.01 as determined by DESeq2. *tetR* is not significantly differentially expressed under any conditions. Note: for DDN032 grown with 1 µg/mL, only two replicates were available for RNA sequencing.

For the lowest tetracycline concentrations considered previously, there is a clear transition into linear growth, so the exponential growth rates reported in Fig. 2B do not correspond to a sustainable growth phenotype. However, it is possible that just below the apparent 2.5 µg/mL threshold, DDN032 may achieve sustained exponential growth at lower-than-wild-type levels. To assess this, we carried out three passages of DDN032 cultures at various tetracycline concentrations including 1.75 µg/mL (Fig. 2C). Even after three passages, exponential growth was observed to be slower in cultures with 1.75 µg/mL than at 5 and 100 µg/mL tetracycline, suggesting that it is possible for true exponential growth to be achieved in an MCR-limited manner (Fig. 2C; Fig. S5).

To test if the growth phenotypes described previously are specific to high energy substrates such as TMA (Fig. 1) and methanol (Fig. 2 to 4), we also conducted growth experiments with DDN032 in acetate minimal media with a series of tetracycline concentrations ranging from 100 µg/mL to 0 µg/mL (Fig. S6). The growth rate of DDN032 was indistinguishable from that of WWM60 at full induction (100 µg/mL), while statistically significant defects were observed at concentrations ≤ 5 µg/mL (Fig. S6). Since we observed no growth defect on methanol for tetracycline concentrations > 2.5 µg/mL, these data suggest that the threshold MCR concentration for growth rate limitation might be substrate-dependent.

### Effect of tetracycline on transcriptome and MCR protein abundance

To quantify MCR repression and the resulting transcriptomic response, we extracted and sequenced total RNA from triplicate cultures grown at various tetracycline concentrations at the timepoint shown in Fig. 2A. This timepoint was chosen as it represented one of the earliest points where the growth curves of the conditions with different concentrations of tetracycline started diverging (Fig. 2A). Transcription of the *mcrA* gene in DDN032 decreased continuously from an FPKM (**F**ragment **P**er **K**ilobase of transcript per **M**illion mapped reads) value of ~850 at 100 µg/mL tetracycline to ~6 at 0 µg/mL (Fig. 3A). Notably, even though no growth difference was observed between WWM60 and DDN032 supplemented with 100 µg/mL tetracycline, there is approximately half the level of *mcrA* transcription in the latter. This inability to reach full induction is possibly because the *tetO1* operator decreases the expression strength of the P*mcrB*(*tetO1*) promoter relative to the native promoter. When plotted against *mcrA* transcript abundance, the growth rates are indistinguishable from the parental strain until transcription decreases to approximately one-third of the parental level, suggesting a large range of MCR-replete growth (Fig. 3B). To assess if this pattern also held true at the protein level, McrA concentration was compared between samples by dilution to extinction on immunoblots, revealing a clear decrease in MCR abundance at lower concentrations of tetracycline for DDN032 (Fig. 3C; Fig S7). Importantly, MCR was found to be in higher abundance in WWM60 than in DDN32 with 5 µg/mL tetracycline, despite the lack of a difference in the growth rate between the two cultures.

At the global level, there is little difference between the transcriptional profile of WWM60 grown with or without tetracycline. Only a single gene was found to be significantly differentially expressed between the two WWM60 conditions, a putative ABC transporter substrate-binding protein (MA0887), indicating that tetracycline alone has little effect on the *M. acetivorans* transcriptome (Fig. 4A and B; Tables S4 and S5). However, the transcriptomic profile of DDN032 was substantially different than that of WWM60 at all investigated tetracycline concentrations (Fig. 4A), and the number of genes differentially expressed between DDN032 and WWM60 increased as tetracycline concentration decreased (Fig. 4B). Principal component analysis revealed that the overall transcriptome structure follows a similar trend as the *mcrA* gene itself (Fig. 4A). Principle component 1, which accounts for 63% of the variance in the transcriptome, falls along a gradient of MCR expression. As expected, the most significantly downregulated genes at 1 and 0 µg/mL tetracycline belong to the *mcr* operon (Fig. 4B). A few other important genes follow the same trend. The expression of *cfbE* (MA3630), the F430 synthetase (25), seems to match that of the *mcr* operon, suggesting the existence of a feedback mechanism to reduce F430 biosynthesis in response to MCR limitation. Similarly, a gradual decrease can be observed in *mmp10* (MA4551), which encodes the protein responsible for the post-translational modification of a conserved arginine residue in MCR (26, 27) (Fig. 4C). Catabolic genes that are highly expressed under normal conditions such as the methanol-specific methyltransferase isoform 1 *mtaCB1* (MA0455–MA0456) and genes of the MTR complex are also significantly decreased in expression upon MCR limitation.

Many genes also respond positively to MCR limitation. The *mtaCB2* (MA4391–MA4392) genes that encode the methanol methyltransferase isoform 2 are typically expressed in the late-exponential or stationary phase. However, during MCR limitation, and the concomitant decrease in *mtaCB1* expression, these genes are strongly upregulated even in the early–mid exponential phase (Fig. 4B and C). Methanol methyltransferase isoform 3 encoded by *mtaBC3* (MA1616–MA1617) follows a similar trend, albeit to a lesser degree. By far, the most dramatic increase in expression occurs in two of the methylsulfide-specific methyltransferases, *mtpCAP* (MA4164–MA4166) and *mtsD* (MA0859), which increase in expression to approximately 100-fold (Fig. 4B and C). Notably, the gene encoding *tetR* does not significantly change in expression level under any of our experimental conditions. This is particularly interesting, given that *tetR* is driven by a p*mcrB* promoter and suggests that the *mcr* operon might be constitutively expressed in the cell.

## DISCUSSION

In this study, we have carried out a comprehensive investigation into the physiological and transcriptomic response of a methanogenic archaeon, specifically to MCR limitation. We find that MCR is not limiting *M. acetivorans* growth in substrate-replete batch cultures, and this observation may explain why some universally conserved MCR-associated proteins can be deleted with little to no effect on growth in this system (10–12). Decreasing MCR can lead to two forms of growth limitation, linear growth under extreme MCR limitation or slower, sustained exponential growth under less drastic limitations (Fig. 2). While this growth-limiting state cannot be maintained indefinitely due to the accumulation of escape mutations, we anticipate that the threshold tetracycline concentration at which a growth defect occurs may be a useful diagnostic feature in assessing the relative fitness of MCR mutants or strains lacking certain conserved MCR-associated proteins. This approach, particularly if coupled with high-throughput growth assays, may enable the screening and initial characterization of many MCR variants, far more than is feasible through existing biochemical approaches.

The global transcriptional response to MCR limitation shared similarities with prior observations of *M. acetivorans* made under various stressful conditions. In particular, the upregulation of the *mtpCAP* operon and *mtsD* is reminiscent of increased methylsulfide production during growth on carbon monoxide (18, 19, 28) and in the absence of HdrABC (29) or pyrrolysine (30). In some of these cases, it has been hypothesized that limitation in MCR activity or change in electron flow results in a buildup of methyl-coenzyme M, which can then be relieved by an increase in the expression of methylsulfide methyltransferases, possibly to help maintain redox balance. While there is no evidence for how this alternate pathway might allow *M. acetivorans* to conserve energy, the results presented here are consistent with the idea that methyl-coenzyme M buildup may induce these alternative methyltransferase systems. It is interesting to note in this context that the MTR complex is significantly downregulated, suggesting that if methyl-coenzyme M build-up is indeed occurring, then the oxidative branch of the methylotrophic pathway is not a viable outlet for these methyl groups, presumably due to a buildup of electron carriers. It is also interesting that while MCR itself may not be actively regulated, as evidenced by the lack of expression change of *tetR*, MCR-associated proteins such as *mmp10* and *cfbE* are downregulated, suggesting a feedback to the expression of MCR-associated proteins. While this trend is not universally true (e.g., *ycaO*, the McrA-glycine thioamidation protein is not significantly regulated), the list of differentially expressed genes presented here may lead to the discovery of additional MCR-related systems of unknown function.

Altogether, we have developed a genetic platform to conclusively demonstrate that MCR does not mediate the rate-limiting step in *M. acetivorans* during routine laboratory growth conditions. While these data are consistent with prior observations from studies with *Methanothermobacter* spp., they diverge from predictions made by metabolic models of *Methanosarcina* spp. Clearly, more physiological studies like ours are required to bridge the gap between research with enzymes in isolation and systems-level analyses of methanogens. While this tool in and of itself will prove to be especially useful to study the properties of MCR mutants and of mutations in MCR-associated proteins, this experimental framework can be expanded to other important enzymes like HdrDE, HdrABC, and MTR to ultimately obtain a robust and quantitative view of methanogenesis.

## MATERIALS AND METHODS

### CRISPR-editing plasmid construction and mutant generation

A target sequence (GTGGACACTTAAAAACGACG) for the *mcrB* promoter in *M. acetivorans* was identified using the CRISPR site finder tool in Geneious Prime version 11.0 with the following parameters: a) an NGG protospacer adjacent motif (PAM) site at the 3’ end and b) no off-target matches allowed. A DNA fragment encoding the single guide RNA (sgRNA) was synthesized as a gblock gene fragment from Integrated DNA Technologies (Coralville, IA, USA) using the target sequence. The sgRNA and a homology repair template to insert the *tetO1* operator site in the promoter of the *mcrBDCGA* operon were cloned into the Cas9 containing vector pDN201, as described previously (31), to generate pGLC001. pGLC001 was digested with PmeI and a repair template introduced, which included the p*mcrB*(*tetO1*) promoter in place of the native *pMcrB* sequence generating pGLC002. The sequences of pGLC001 and pGLC002 were verified by Sanger sequencing at the Barker sequencing facility at the University of California, Berkeley. A cointegrate of pGLC002 and pAMG40 was generated using the Gateway BP Clonase II Enzyme mix as per the manufacturer’s instructions (Thermo Fisher Scientific, Waltham, MA, USA) and named pGLC003. All *E. coli* transformations were conducted with WM4489 (32), as described previously.

A 10-mL culture of *M. acetivorans* in high salt (HS) medium with 50 mM trimethylamine (TMA) in the late-exponential phase was used for liposome-mediated transformation with pGLC003, as described previously (33). Transformants were plated in agar solidified HS medium with 50 mM TMA, 100 µg/mL tetracycline, and 2 µg/mL puromycin and incubated in an anerobic incubator located inside the anerobic chamber at 37 ˚C with H_2_S/CO_2_/N_2_ (1,000 ppm/20%/balance) in the headspace. Colonies were screened for the mutation and sequence-verified by Sanger sequencing at the Barker sequencing facility at the University of California, Berkeley. Several colonies that tested positive for the desired mutation were streaked out on HS medium with 50 mM TMA, 100 µg/mL tetracycline, and 20 µg/mL 8ADP to cure the mutagenic plasmid. Plasmid-cured mutants were verified by screening for the absence of the *pac* gene present on the plasmid with PCR. A single isolate of the plasmid-cured mutant was grown in liquid culture with 50 mM TMA and 100 µg/mL tetracycline and saved as DDN032. All primers, plasmids, and strains used in this study are listed in Tables S1 and S3, respectively.

### Growth measurements

All growth experiments were conducted using either WWM60 (*M. acetivorans* ∆*hpt*:: P*mcrB-tetR*) (23) or DDN032 [WWM60-P*mcrB*(*tetO1*)-*mcrBDCGA*], a strain with the chromosomal *mcr* genes containing a *tetO1* operator site inserted in the promoter. All growth analyses were conducted in 10 mL of high salt (HS) media containing methanol (125 mM), TMA (50 mM), sodium acetate (20 mM) as a carbon source, and a pressurized CO_2_/N_2_ (20:80) headspace, as previously described (34). Various concentrations of tetracycline were added to the media, as indicated, requiring the media to be protected from light to prevent degradation. Anerobic tetracycline stocks were prepared using tetracycline hydrochloride (Millipore Sigma, Bulington, MA, USA; Product number T7660) fresh in anerobic water on the day of the inoculation, as described previously (23).

All *M. acetivorans* growth rates were determined by measuring the optical density (at 600 nm) of cultures grown in Balch tubes containing 10 mL HS media with media additions as indicated. All optical density measurements were made using a UV–Vis spectrophotometer (Genesys50, Thermo Fisher Scientific, Waltham, MA, USA). Growth rates were determined using the best fit line of the log_2_-transformed optical density data with maximal R^2^ values.

### DNA extraction and sequencing

Genomic DNA was extracted from a 10-mL late-exponential phase culture of DDN032 in HS medium with 125 mM methanol and 100 µg/mL tetracycline as well as the escape mutant in HS medium with 125 mM methanol using a Qiagen Blood and Tissue Kit as per the manufacturer’s instructions (Qiagen, Hilden Germany). Library preparation and Illumina sequencing (150-bp paired-end reads) were conducted at Seqcenter (Pittsburgh, PA). The sequencing reads were mapped to the *M. acetivorans* C2A reference genome using breseq version 0.38.1 with default parameters (35).

### RNA extraction, sequencing, and transcriptomic analysis

Quintuplicate cultures of DDN032 and WWM60 were grown in 10 mL HS medium with 125 mM methanol and different concentrations of tetracycline, as indicated in the text. A 3-mL culture was removed for RNA extraction at an optical density between 0.2 and 0.6. The culture was immediately mixed at a ratio of 1:1 with RNA*later*, centrifuged at 10,000 x g for 10 minutes at 4°C, and the resulting pellet was applied to a Qiagen RNeasy Mini Kit (Qiagen, Hilden, Germany) and RNA extraction proceeded according to the manufacturer’s instructions. DNAse treatment, rRNA depletion, cDNA preparation, and Illumina library preparation and sequencing were performed at SeqCenter (Pittsburgh, PA). Analysis of transcriptome data was carried out on the Kbase bioinformatics platform using default parameters for Bowtie2, Cufflinks, and DESeq2 (36). Briefly, raw reads were mapped to the *M. acetivorans* WWM60 genome using Bowtie2 (37), assembled using Cufflinks (38), and fold changes and significances values were calculated with DESeq2 (39).

### Immunoblot analysis of McrA

Late exponential-phase cultures were harvested by centrifugation, resuspended in 1 mL of lysis buffer (50 mM NaH_2_PO_4_, 2 U/mL DNase I, 1 mM phenylmethylsulfonyl fluoride) and incubated at room temperature for 10 minutes. An appropriate volume of a 5 M NaCl stock solution was added to bring the lysate to a final concentration of 300 mM NaCl. The lysate was then cleared by centrifugation [>10,000 rcf, 4°C, 30 m, Sorvall Legend XTR (Thermofisher, Waltham, MA)], and the decanted supernatant was quantified with a microplate Bradford assay as per the manufacturer’s instructions (Sigma-Aldrich, Sant Louis, MO, USA).

Dilution series containing equal amounts of protein were prepared and separated on 12% Mini-PROTEAN TGX gels (BioRad, Hercules, CA, USA) by SDS-PAGE and then transferred onto 0.2-µM polyvinylidene difluoride (PVDF) membranes using the Trans-Blot Turbo system (BioRad) using Trans-Blot Turbo 0.2 PVDF transfer packs as per the manufacturer’s instructions. The membranes were then washed with phosphate-buffered saline containing 0.05% (v/v) Tween-20 (PBST) for 5 minutes at room temperature. Nonspecific binding was blocked by incubating in PBST containing 5% (w/w) nonfat milk powder for 1 hour at room temperature and washing four times lasting 5 minutes each in PBST. The membranes were then incubated overnight at 4°C in PBST with polyclonal rabbit antibodies raised against McrA (1:10000 dilution) (GenScript, Piscataway, NJ, USA), washed four times for 5 minutes in PBST, and then incubated with anti-rabbit horseradish peroxidase (HRP) conjugate antibodies (1:20000 dilution) (Promega, Madison, WI, USA) for 2 hours at room temperature. Following four additional 5-minute washes in PBST and three final washes in phosphate-buffered saline without Tween-20, the membranes were developed with a 5-minute incubation in Immobilon Western Chemiluminescent HRP substrate (EMD Millipore, Burlington, MA, USA) and imaged using a ChemiDoc XRS+ (BioRad). Sixty images were collected over 1 minute of imaging, and the final images, which lacked oversaturation on any target bands, were selected for analysis using Image Lab.

## Data Availability

Raw reads have been deposited in the Sequence Read Archive (SRA) and can be accessed under BioProject numbers PRJNA800036 and PRJNA1107246.

## SUPPLEMENTAL MATERIAL

Supplemental material aem.02220-23-s0001.docx Tables S1 to S3; Fig. S1 to S7.

ASM does not own the copyrights to Supplemental Material that may be linked to, or accessed through, an article. The authors have granted ASM a non-exclusive, world-wide license to publish the Supplemental Material files. Please contact the corresponding author directly for reuse.
