# Supplementary material for: Physiological and transcriptomic response to methyl-coenzyme M reductase limitation in Methanosarcina acetivorans
Source: Appl Environ Microbiol. 2024 Jun 25;90(7):e02220-23. doi: 10.1128/aem.02220-23 (PMC11267899; doi:10.1128/aem.02220-23)
Supplement: Supplemental material — Tables S1 to S3; Fig. S1 to S7. [file aem.02220-23-s0001.docx]

**Supplementary Material**

Physiological and transcriptomic response to methyl-coenzyme M reductase limitation in *Methanosarcina acetivorans*

Grayson L. Chadwick^a^, Gavin A. Dury^a^, Dipti D. Nayak^a, #^

Department of Molecular and Cell Biology, University of California, Berkeley, California, USA^a^

Running Head: Methanogens produce excess MCR under optimal conditions

#Address correspondence to:

Dipti D. Nayak ([dnayak@berkeley.edu](mailto:dnayak@berkeley.edu))

Department of Molecular and Cell Biology, 1 Barker Hall #3204, University of California, Berkeley, CA 94720-3204

Tel: 510-664-5267

**Supplementary Table 1: List of primers used in this study**

| **Primer** | **Sequence** | **Description** | **Reference** |
| --- | --- | --- | --- |
| GLC001 | AGTGTCTGACACAGTAG | Foward primer for generating ~1kilobase (kb) *of* *mcrB* from *Methanosarcina acetivorans* for repair template | This study |
| GLC002 | GATGTTGTTCCTGCAGGTTT  AAGTACAGAAGTGTTGAG | Reverse primer for generating ~1kb of *mcrB* from *M. acetivorans* for repair template (region in red is a 20bp overhang for Gibson assembly into *Pme1* digested pGLC001) | This study |
| GLC003 | GCCTTTTTTTTTCGAAGTTT  CAATTTCAGTAAATTCGGAT | Foward primer for generating ~1kb of upstream region *of* *mcrB* from *M. acetivorans* for repair template (region in red is a 20bp overhang for Gibson assembly into *Pme1* digested pGLC001) | This study |
| GLC004 | TCATGGATTTTTTTAAAAATCATT | Reverse primer for generating ~1kbp of upstream region of McrB from *M. acetivorans* for repair template | This study |
| GLC005 | GTCTACTGTGTCAGACACT | Reverse primer for sequencing gblock of pMcrB(tetO1) | This study |
| GLC006 | TTTTCCTCTGTCGTCGTAG | Reverse primer for sequencing gblock of pMcrB(t*etO1*) inside pGLC002 | This study |

**Supplementary Table 2: List of plasmids used in this study**

| **Plasmid** | **Description** | **Antibiotic Resistance** | **Reference** |
| --- | --- | --- | --- |
| pDN201 | Cas9 from pMJ806 in place of *uidA* in pJK027A cloned in using Gibson assembly | Chloroamphenicol | (1) |
| pAMG40 | E. coli-Methanosarcina shuttle vector for fosmid retrofitting encoding ampicillin resistance and lambda attB | Kanamycin | (2) |
| pGLC001 | pDN201 cut with *AscI* and insertion of a guide construct for cutting *mcrB* promoter region | Chloroamphenicol | This study |
| pGLC002 | pGLC001 cut with PmeI to insert the repair template for *PmcrB(tetO1)* | Chloroamphenicol | This study |
| pGLC003 | Cointegrate of pGLC002 and pAMG40 obtained by Gateway cloning (BP clonase) | Chloroamphenicol/  Kanamycin | This study |

**Supplementary Table 3: List of strains used in this study**

| **Strain** | **Plasmid** | **Antibiotic Resistance** | **Genotype** | **Reference** |
| --- | --- | --- | --- | --- |
| WM7959 | pDN201 | Chloroamphenicol | WM4489/pDN201 | (1) |
| WM3357 | pAMG40 | Kanamycin | WM1788/pAMG40 | (2) |
| DN112 | pGLC001 | Chloroamphenicol | WM4489/pGLC001 | This study |
| DN113 | pGLC002 | Chloroamphenicol | WM4489/pGLC002 | This study |
| DN124 | pGLC003 | Chloroamphenicol/  Kanamycin | WM4489/pGLC003 | This study |


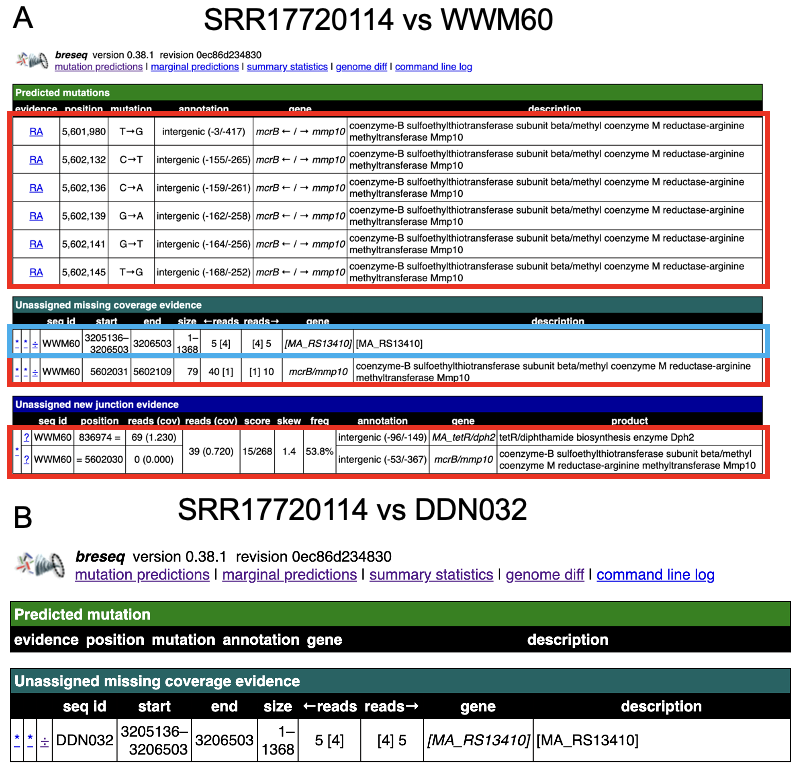


**FIG S1**: Genome resequencing. A) BreSeq analysis of DDN032 (SRR17720114) raw reads vs. WWM60. Changes in red correspond to expected modifications from inserting the *tetO1* operator site at the *mcr* promoter. Note: “Unassigned new junction evidence” is a spurious call due to the similarity between the native *mcr* promoter and the *mcr* promoter used to drive the t*etR* gene (MA_tetR). The missing coverage evidence for MA_RS13410 corresponds to a highly active transposase which has identical sequences spread throughout the genome often causing spurious missing coverage calls. B) BreSeq analysis of DDN032 (SRR17720114) raw reads vs. the expected full genome sequence of DDN032 shows no mutations and just the aforementioned low coverage of a transposase sequence.


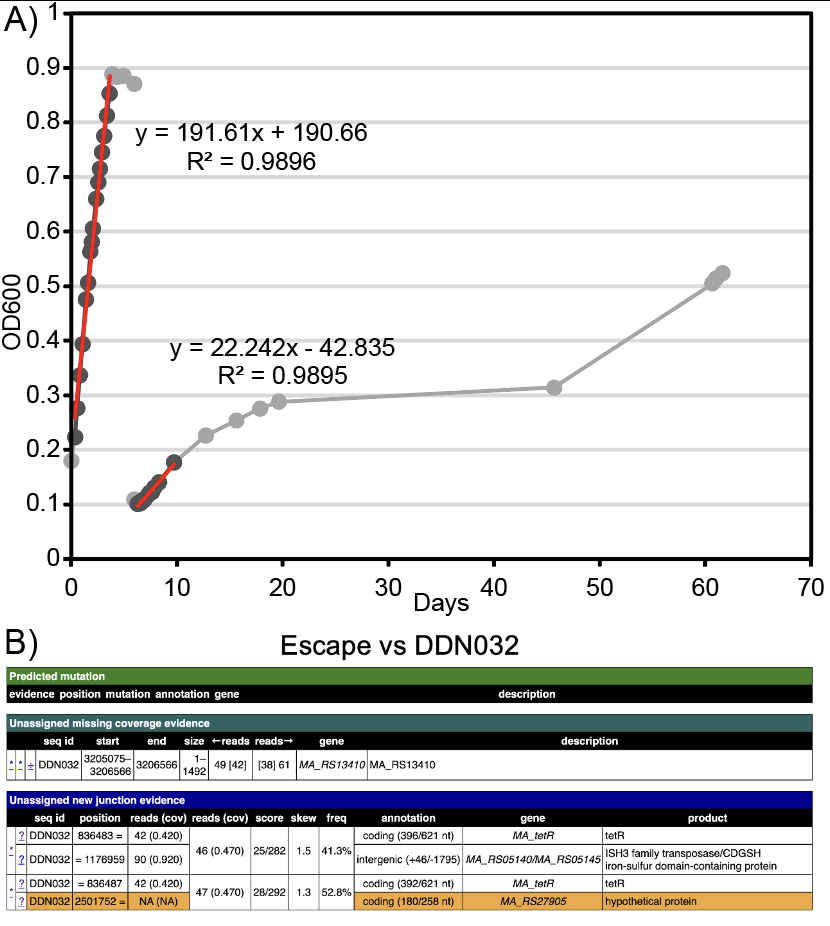


**FIG S2**: Long term incubation of DDN032 without tetracycline selects for escape mutants that inactivate the tetracycline repressor and allow for the expression of the *mcr* operon in the absence of tetracycline. A) Growth curves of DDN032 after washing and inoculation into tetracycline free media is shown on the left and a subsequent passage that leads to slow linear growth is shown on the right. This slower linear growth is consistent similar experiments shown in **Fig. 1C**. The slow linear growth levels off after 20 days of inoculation and somewhere beteen 45 and 60 days, significant additional growth occurrs, yielding a strain capable of exponential growth without the addition of tetracycline (i.e. an escape mutant). B) Whole genome sequencing of this escape mutant revealed the introduction of a transposon into the *tetR* gene as indicated by the presence of two new junction between the *tetR* gene and an ISH3 family transposase.


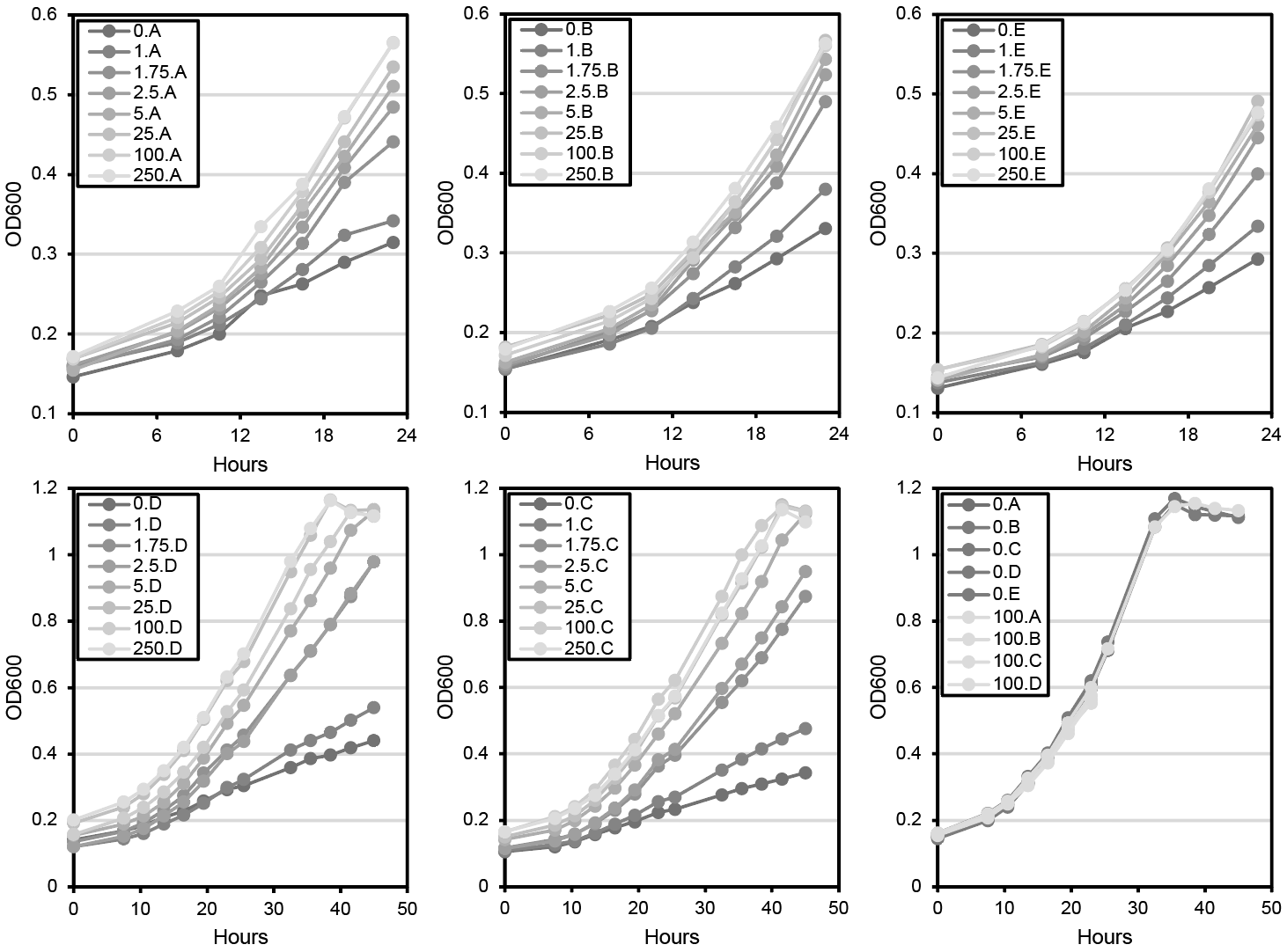


**FIG S3**: All individual growth curves from the experiment conducted for **Fig 2A-B**. The first five panels show replicates A-E of DDN032 at various tetracycline concentrations as indicated in the figure legend. Replicates A, B and E were harvested 24 hours after inoculation for RNA sequencing (see data in **Fig 3-4**), whereas replicates C and D were allowed to continue growth. The final panel shows all growth curves of WWM60, three of which were also harvested at 24 hours for RNA sequencing (see data in **Fig 3-4**). Legends show the concentration of tetracycline in µg/ml.


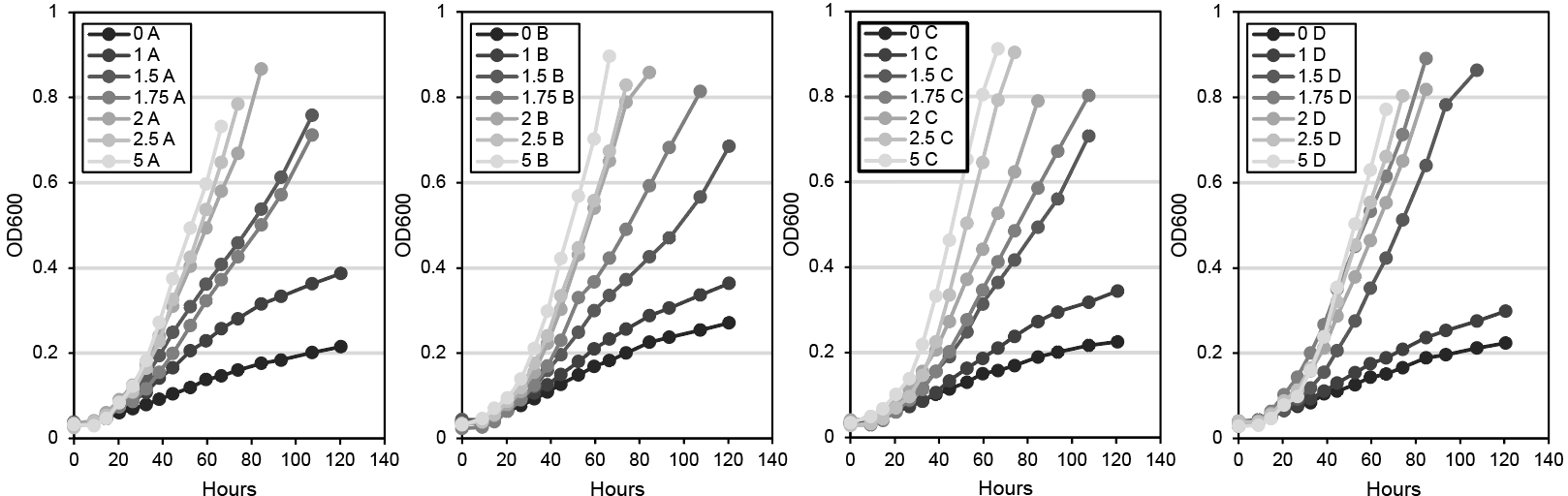


**FIG S4**: All growth curves from Experiment 2 shown in **Fig 2A-B**. Legends show the concentration of tetracycline in µg/ml.


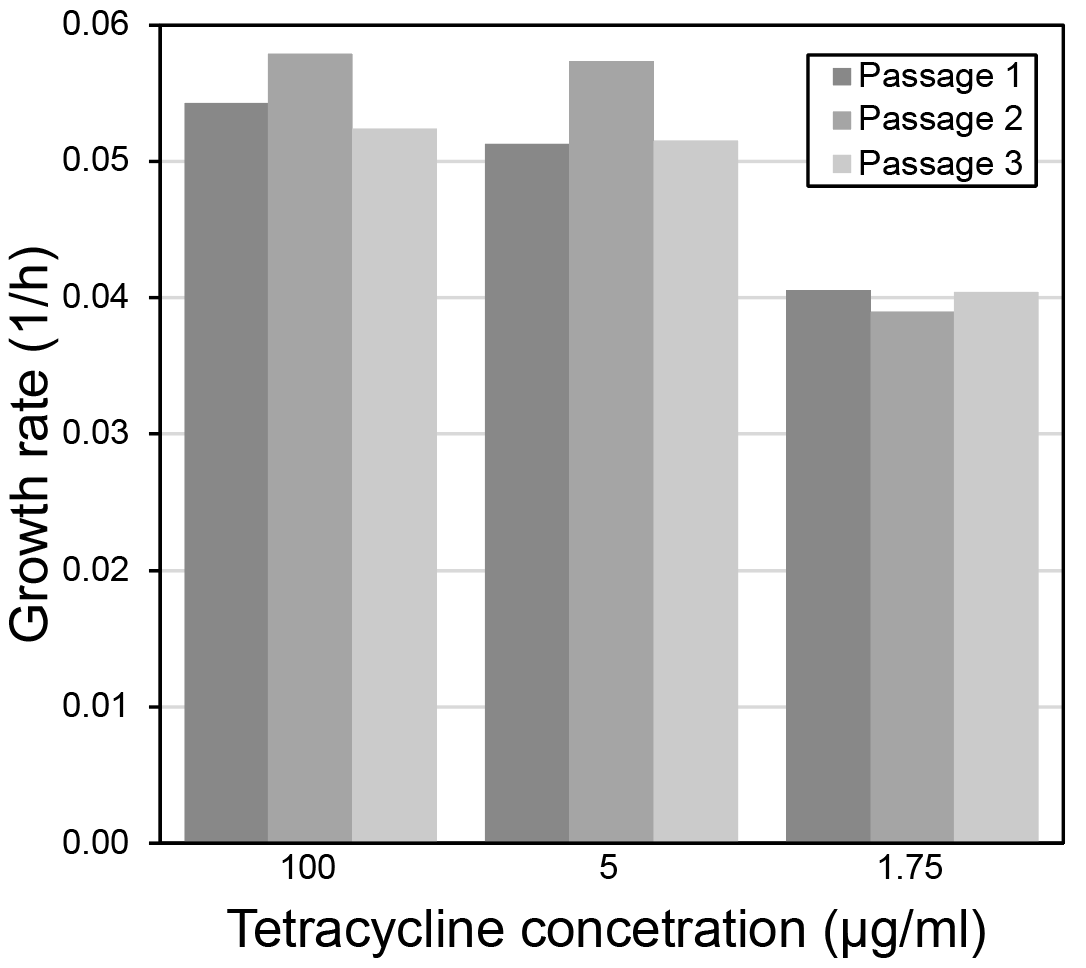


**FIG S5**: Growth rates for the exponential growth of sequential passages of DDN032 shown in **Fig 2C**.

**
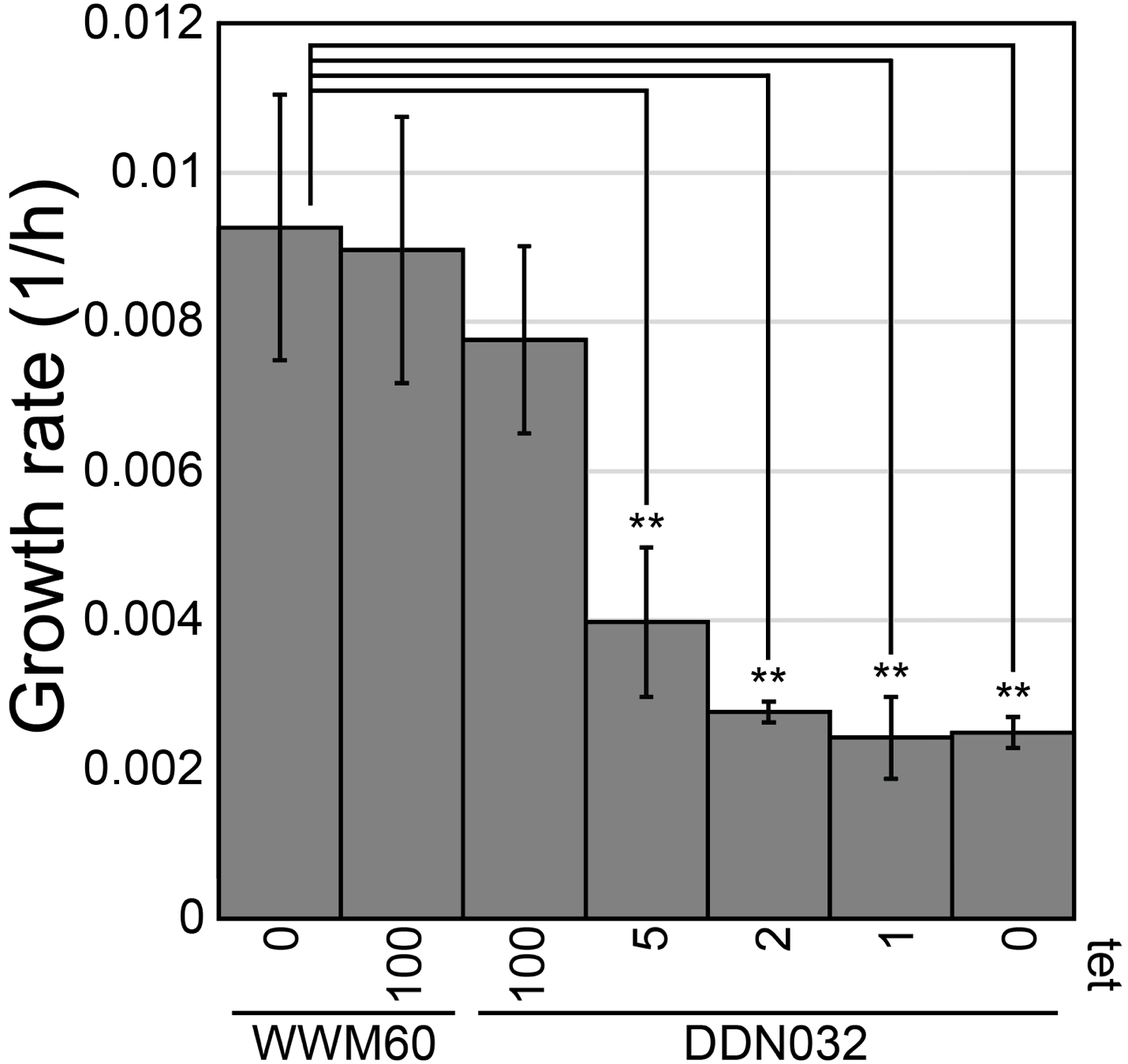
**

**FIG S6**: Growth rates for DDN032 and WWM60 grown on high salt acetate media with various tetracycline concentrations. Growth rates that are significantly different than WWM60 with 0 µg/ml tetracycline by ANOVA and Tukey’s Honest Significant Difference test are indicated (** p-value ≤ 0.01). Error bars represent standard deviations of biological triplicates.

**FIG S7**: Representative full images of anti-McrA western blots. Comparisons between Mcr abundance at different levels of tetracycline concentrations (listed below each set of bands) should only be made with bands resulting from the same total protein load (μg) (listed above each band) on the same blot. Comparisons between blots is not possible. In addition to the 60 kDa band, associated with functional McrA, a secondary band at 50 kDa was detected. This band was confirmed by mass spectrometry to also be McrA and is presumed to be a degradation product, as has been observed in previous studies (3). The apparent high density of the secondary band in the 0 μg/mL tetracycline samples may be explained by the very low number of generations this culture completed before harvesting; these bands may represent the original pool of Mcr from the fully induced seed culture.

**References**

1. Nayak DD, Metcalf WW. 2017. Cas9-mediated genome editing in the methanogenic archaeon *Methanosarcina acetivorans*. Proc Natl Acad Sci USA 114:2976–2981.
2. Guss AM, Rother M, Zhang JK, Kulkarni G, Metcalf WW. 2008. New methods for tightly regulated gene expression and highly efficient chromosomal integration of cloned genes for *Methanosarcina* species. Archaea 2:193–203.
3. Aldrich HC, Beimborn DB, Bokranz M, Schönheit P. 1987. Immunocytochemical localization of methyl-coenzyme M reductase in *Methanobacterium thermoautotrophicum.* Arch Microbiol 147:190-194.
